# Unique Self-Phosphorylating Polybenzimidazole of the 6F Family for HT-PEM Fuel Cell Application

**DOI:** 10.3390/ijms25116001

**Published:** 2024-05-30

**Authors:** Igor I. Ponomarev, Yulia A. Volkova, Kirill M. Skupov, Elizaveta S. Vtyurina, Ivan I. Ponomarev, Mikhail M. Ilyin, Roman Y. Nikiforov, Alexander Y. Alentiev, Olga M. Zhigalina, Dmitry N. Khmelenin, Tatyana V. Strelkova, Alexander D. Modestov

**Affiliations:** 1 A.N. Nesmeyanov Institute of Organoelement Compounds of Russian Academy of Sciences, 28 Vavilova St., bld. 1, Moscow 119334, Russia; gagapon@ineos.ac.ru (I.I.P.); yvolk@ineos.ac.ru (Y.A.V.); ves1809@yandex.ru (E.S.V.); ivan.ponomarev84@gmail.com (I.I.P.); kotosok@yandex.ru (M.M.I.); tv.strelkova@yandex.ru (T.V.S.); 2 A.V. Topchiev Institute of Petrochemical Synthesis, Russian Academy of Sciences, 29 Leninsky Av., Moscow 119991, Russia; nru@ips.ac.ru (R.Y.N.); alentiev@ips.ac.ru (A.Y.A.); 3 A.V. Shubnikov Institute of Crystallography of Federal Scientific Research Centre “Crystallography and Photonics”of Russian Academy of Sciences, 59 Leninsky Av., Moscow 119333, Russia; zhigal@crys.ras.ru (O.M.Z.); xorrunn@gmail.com (D.N.K.); 4 A.N. Frumkin Institute of Physical Chemistry and Electrochemistry, Russian Academy of Sciences, 31 Leninsky Av., bld. 4., Moscow 119071, Russia

**Keywords:** polybenzimidazole 6F, polyamide 6F, polymer-electrolyte membrane, fuel cell, HT-PEM, phosphorylation, proton conductivity, membrane–electrode assembly, carbon nanofibers, platinum nanoparticles

## Abstract

High-temperature polymer-electrolyte membrane fuel cells (HT-PEMFCs) are a very important type of fuel cells since they operate at 150–200 °C, making it possible to use hydrogen contaminated with CO. However, the need to improve the stability and other properties of gas-diffusion electrodes still impedes their distribution. Self-supporting anodes based on carbon nanofibers (CNF) are prepared using the electrospinning method from a polyacrylonitrile solution containing zirconium salt, followed by pyrolysis. After the deposition of Pt nanoparticles on the CNF surface, the composite anodes are obtained. A new self-phosphorylating polybenzimidazole of the 6F family is applied to the Pt/CNF surface to improve the triple-phase boundary, gas transport, and proton conductivity of the anode. This polymer coating ensures a continuous interface between the anode and proton-conducting membrane. The polymer is investigated using CO_2_ adsorption, TGA, DTA, FTIR, GPC, and gas permeability measurements. The anodes are studied using SEM, HAADF STEM, and CV. The operation of the membrane–electrode assembly in the H_2_/air HT-PEMFC shows that the application of the new PBI of the 6F family with good gas permeability as a coating for the CNF anodes results in an enhancement of HT-PEMFC performance, reaching 500 mW/cm^2^ at 1.3 A/cm^2^ (at 180 °C), compared with the previously studied PBI-O-PhT-P polymer.

## 1. Introduction

One of the most significant challenges in the field of renewable energy is the development of highly efficient and long-lasting hydrogen–air fuel cells (FCs) based on a polymer-electrolyte (proton-exchange) membrane (PEM). These types of fuel cells directly convert chemical energy from an oxidation reaction into electrical energy using hydrogen or other low-boiling organic fuels [1,2]. Among the different FC types, the high-temperature polymer-electrolyte membrane fuel cell (HT-PEMFC) [3,4,5,6,7,8,9,10,11,12,13,14,15] based on a polybenzimidazole (PBI) proton-conducting membrane offers several unique advantages and holds significant potential for widespread distribution. Many studies have been conducted on the feasibility of using PBI-based PEMs for HT-PEMFC in heavy vehicles [4,15]. As part of this study, a priority task was identified to increase the power of this type of fuel cell. At the same time, a necessary condition for achieving this goal is the improvement of the most significant components of the HT-PEMFC, specifically, a benzimidazole proton-conducting membrane, gas-diffusion electrodes, and a platinum composite electrocatalyst.

**Figure 1:**
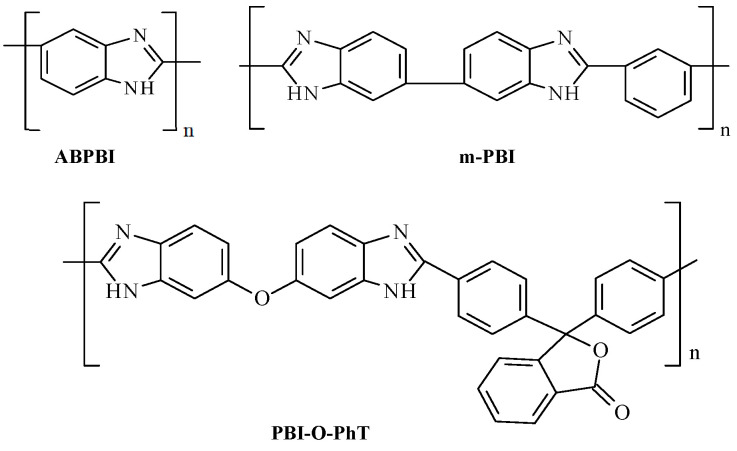
Chemical structures of ABPBI, *m*-PBI, and PBI-O-PhT.

The PBI-based PEMs for HT-PEMFC should be able to operate under corrosive phosphoric acid (PA) conditions at 150–200 °C for several thousand hours, i.e., under rather harsh conditions. The most extensively studied PBI is the commercially available poly-2,2′-(*m*-phenylene)-5,5′-bibenzimidazole (*m*-PBI, Celazole^®^ (PBI Performance Products, Charlotte, NC, USA), Figure 1) created by Vogel and Marvel in 1961 [16].

**Figure 2:**
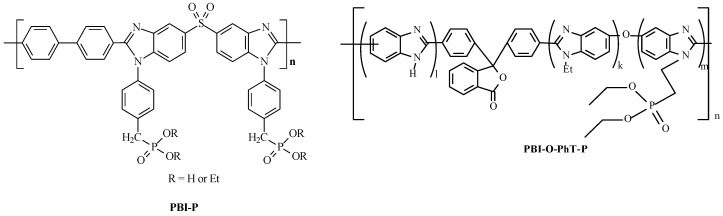
Chemical structures of PBI-P and PBI-O-PhT-P.

Many other PBIs have also been synthesized. Among them, the cheapest and most available ABPBI [1,3,4], as well as the widely studied cardo PBI-O-PhT [17,18,19], are the most interesting. In the studies of PBI membranes of different structures, it has been found that traditional PBI membranes become involved in the process of PA leaching due to water generation in the FC electrochemical reaction. Therefore, attention has been paid to the synthesis of phosphorylated PBIs (PBI-P, PBI-O-PhT-P), which contain phosphoryl groups in their side chains and are able to maintain the proton conductivity of the membranes during long-term FC operation (Figure 2).

**Figure 3:**
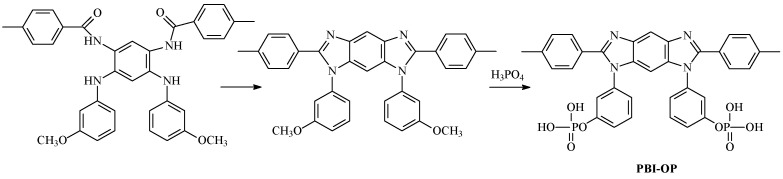
Obtaining of self-phosphorylated PBI.

Some of such polymers have been described in the literature [20,21,22]. Among the latest developments in the field of PBI synthesis, a novel approach to the preparation of functionalized PBI should be noted. Particularly, the process occurs via a step in the polyamidation of functionalized tetraamines followed by polyheterocyclization into the required PBIs [22]. This approach has been developed in [23], where self-phosphorylating PBIs (PBI-OP) are obtained via the polyamidation of methoxyl derivatives of tetramines (Figure 3).

Such an approach does not require the synthesis of expensive and hard-to-reach phosphorus-containing monomers. In terms of power characteristics, the membrane–electrode assembly (MEA) with the PBI-OP membrane and Celtec^®^-P1000 electrodes outperformed the commercially available analog with the Celazole^®^ membrane, and the maximum peak power achieved was 680 mW/cm^2^ at 180 °C. This two-step synthesis of novel PBIs, involving the formation of a prepolymer (fore-polyamide) at room temperature followed by thermal cyclization, addresses several environmental challenges associated with the production of industrial membranes from PBI and significantly reduces the cost of producing proton-conducting membranes for hydrogen/air HT-PEMFCs.

As previously discussed, a prerequisite for achieving the objective is the enhancement of all key components of the HT-PEMFC: a benzimidazole proton-conducting membrane, gas-diffusion electrodes (GDEs), and a platinum composite. Earlier, we proposed a fundamentally new approach to obtain gas-diffusion electrodes for HT-PEMFC [24]. The process involves the application of self-supported (self-standing) GDEs (anodes and/or cathodes), which are based on carbon nanofibers (CNFs) produced by electrospinning polyacrylonitrile solution containing a zirconium salt, followed by pyrolysis. After the deposition of Pt nanoparticles on the CNF surface, zirconium-containing composite GDEs are produced. In order to improve the triple phase boundary and proton conductivity of the anode, make a continuous interface with a proton-conducting membrane, and attain higher HT-PEMFC performance, three polymers were tested as surface modifiers for the CNF anode with deposited Pt: Nafion^®^ (DuPont, Wilmington, DE, USA), PIM-1, and the abovementioned PBI-O-PhT-P polymer (Figure 2) [25]. The performance of HT-PEMFC with the PBI-O-PhT-P-coated anode (PBI-O-PhT-P/Pt/CNF) was significantly higher than in the case of the uncoated Pt/CNF sample. The application of Nafion^®^ and PIM-1 to the already prepared Pt/CNF resulted only in a slight decrease in HT-PEMFC performance, making it comparable to the uncoated Pt/CNF sample.

It is well established that fluorinated aromatic polymers featuring a rigid kink 6F structure exhibit enhanced chemical stability, heat resistance, hydrophobicity, and increased gas permeability [26,27]. PBIs based on 6F monomers have been found to exhibit exceptionally high permeability for small gas molecules, as well as excellent molecular sieving properties [28,29].

The aim of this study is to enhance the efficiency of the surface treatment of HT-PEMFC Pt/CNF anodes (and their performance in FC) using a novel self-phosphorylating PBI containing hexafluoroisopropylidene (6F) fragments applied as a surface modifier.

## 2. Results and Discussion

**Figure 4:**
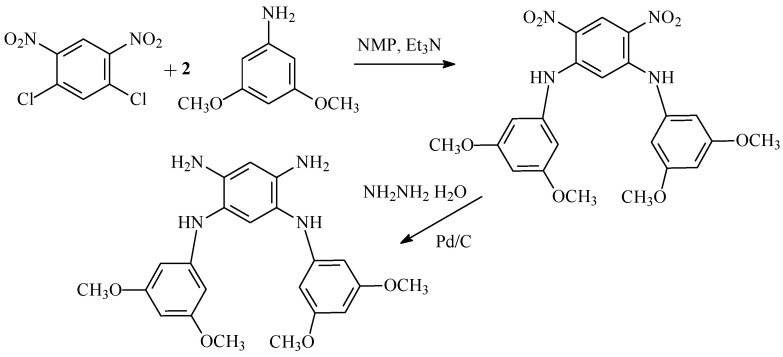
Synthesis of *N*^1^,*N*^5^-bis(3,5-dimethoxyphenyl)-1,2,4,5-benzenetetramine.

A new tetramine, *N*^1^,*N*^5^-bis(3,5-dimethoxyphenyl)-1,2,4,5-benzenetetramine, was obtained from 3,5-dimethoxyaniline and 1,5-dichloro-2,4-dinitrobenzene with a yield of 85% according to the scheme described in [23] (Figure 4).

**Figure 5:**
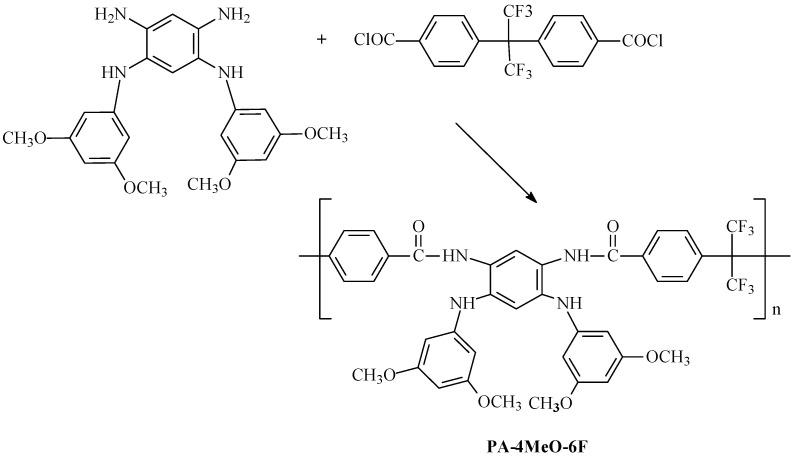
The obtaining of PA-4MeO-6F via the polyamidation process.

The structure of *N*^1^,*N*^5^-bis(3,5-dimethoxyphenyl)-1,2,4,5-benzenetetramine is confirmed using ^1^H NMR and elemental analysis. The ^1^H NMR spectrum is provided in Figure S1. The polyamidation of *N*^1^,*N*^5^-bis(3,5-dimethoxyphenyl)-1,2,4,5-benzenetetramine and 4,4′-(hexafluoroisopropylidene)bis(benzoic acid chloride) was carried out in amide-type solvents (DMA, DMA/Et_3_N, NMP) under different conditions with the aim of obtaining a high-molecular-weight product, namely, PA-4MeO-6F (Figure 5).

Additional information on PA-4MeO-6F synthesis is provided in Table 1. Gel permeation chromatography (GPC) curves for PA-4MeO-6F are provided in Figures S2 and S3.

As a result of the polycondensation process, viscous solutions are always formed. Upon dilution, they precipitate by alcohol in the form of fine fibers. The molecular weights of the PA-4MeO-6F samples were determined using viscosimetry and gel permeation chromatography (GPC) (Table 1). The synthesized polyamides can be dissolved in amide-type solvents, DMSO, hexafluoroisopropanol, and a mixture of chloroform and trifluoroacetic acid; moreover, they possess film-forming properties.

In Figures S4 and S5, the ^1^H and ^19^F NMR spectra of PA-4MeO-6F in DMSO-d6 are shown. The spectra fully correspond to its structure.

**Figure 6:**
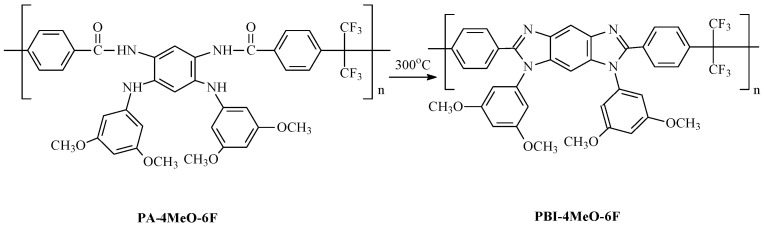
The obtaining of PBI-4MeO-6F via thermal heterocyclization.

Based on the PA-4MeO-6F polymer obtained from the NMP solvent (Table 1), a durable and sufficiently elastic film was prepared. The mechanical properties were as follows: tensile strength of 70 ± 5 MPa, elongation at break of 8 ± 2%, and Young’s modulus of 1300 ± 100 MPa. The PA-4MeO-6F film was introduced into thermal heterocyclyzation for 1 h under vacuum at 300 °C with the formation of PBI-4MeO-6F functionalized with four methoxy groups (Figure 6).

**Figure 7:**
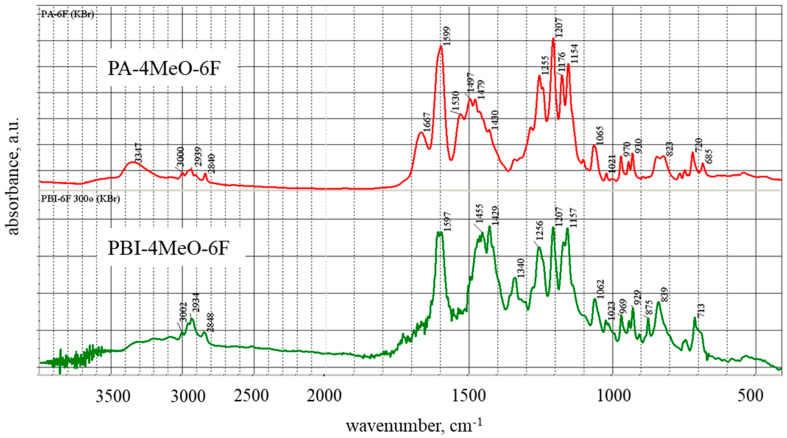
FTIR spectra for PA-4MeO-6F (red, **top**) and PBI-4MeO-6F (green, **bottom**).

The process of thermal cyclization for PA-4MeO-6F to PBI-4MeO-6F was controlled using FTIR spectroscopy (Figure 7).

The absorption bands of the amide groups, amide I (1667 cm^−1^) and amide II (1530 cm^−1^), as well as those in the region between 3200 and 3400 cm^−1^, which are attributed to the NH groups, completely disappeared [30].

**Figure 8:**
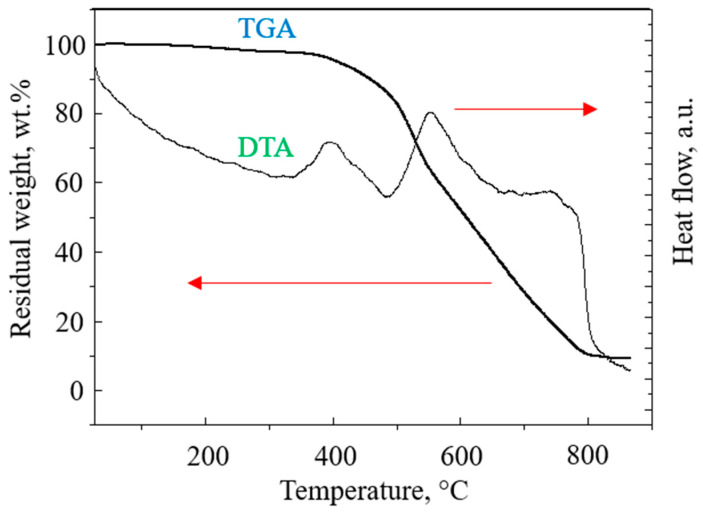
TGA and DTA for the PBI-4MeO-6F polymer.

The PBI-4MeO-6F films after thermal cyclization possess a sufficient tensile strength of 75 ± 5 MPa, an elongation at break of 8 ± 2%, and a Young’s modulus of 1500 ± 100 MPa. According to the thermogravimetric analysis (TGA) and differential thermal analysis (DTA) in air, the polymer possesses high heat resistance and begins to decompose at 407 °C (5 wt.% of loss weight). A more intensive decomposition occurs at >550 °C (Figure 8). It can be seen from the DTA data that the decomposition occurs in two steps. Most probably, the first step of decomposition is related to the loss of OMe-groups, and the second step is related to the thermooxidation of the aromatic part of PBI.

It is important to note that, after the thermal cyclization of the PA-4MeO-6F polyamide film into PBI-4MeO-6F, the polymer completely loses its solubility in phosphoric acid, thereby avoiding the poisoning of platinum. The cross-linking of PBI-4MeO-6F during the polymerization process ensures that the polymer coating remains attached to the surface of the nanofibers even at elevated temperatures (150–200 °C) [31]. The possibility of obtaining the PBI-4MeO-6F films allows us to assess the gas permeability of these films for hydrogen and oxygen, as well as to compare them to the most effective surface modifier of the Pt/CNF anode, PBI-O-PhT-P (Figure 2), which was found earlier [25]. The hydrogen permeability of PBI-4MeO-6F (Table 2) is 41.1 Barrer, which is 4.25 times higher than in the case of the PBI-O-PhT-P film; moreover, it allows for a more efficient formation of the triple-phase boundary.

Additionally, it should be noted that permeability for the oxygen of the PBI-4MeO-6F film is considerably higher: 7.15 vs. 0.88 Barrer, i.e., 8.13 times higher than for the PBI-O-PhT-P film. Low permeabilities for nitrogen, 1.97, and even 0.22 Barrer for PBI-4MeO-6F and PBI-O-PhT-P, respectively, could potentially play a significant role if the polymers are applied on the cathode side. In this case, supplied air could become “enriched” with oxygen when Pt electrocatalyst particles (due to the difference in O_2_ and N_2_ permeabilities) are reached, resulting in higher FC performance. This makes the novel PBI-4MeO-6F promising for the surface modification of CNF electrodes while also ensuring efficient proton transport across the entire surface of CNF without hindering the access of gases to the nanocrystalline Pt catalyst surface.

**Figure 9:**
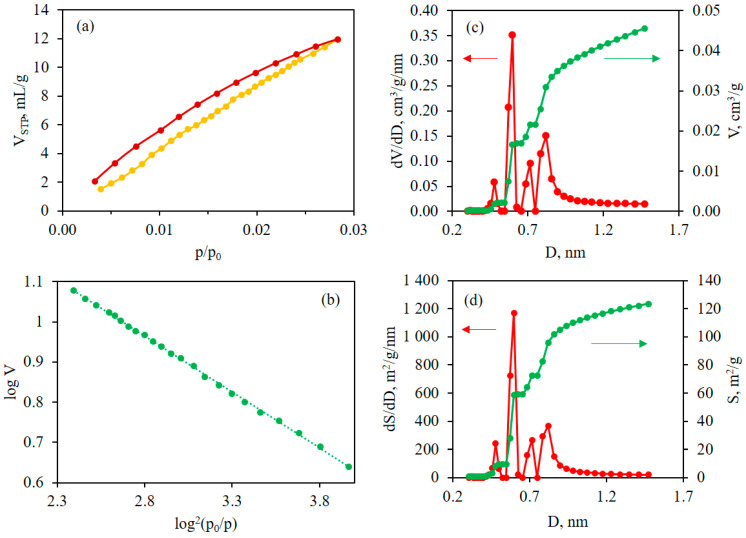
(**a**) Sorption (yellow) and desorption (dark red) isotherms; (**b**) DR plot; (**c**) NL-DFT integral (green) and differential (red) pore size distribution dV/dD vs. pore size; (**d**) NL-DFT integral (green) and differential (red) pore size distribution dS/dD vs. pore size.

In order to confirm the promising gas permeability characteristics of the PBI-4MeO-6F films, a study was carried out to examine their porometric properties and evaluate their microporosity. The CO_2_ sorption method (273 K) is widely applied for studies of microporosity since it allows more micropores to be observed due to the ability of CO_2_ molecules to penetrate micropores at 273 K, which is better than N_2_ molecules at 77 K (because of kinetic reasons) [33]. This approach allows the calculation of the specific volume (SV) and the adsorption energy of micropores according to the Dubinin–Radushkevich (DR) method. The implementation of the non-local density functional theory (NL-DFT) method to the obtained CO_2_ sorption isotherm allows the calculation of SV and specific surface area (SSA) for micropores. In Figure 9a, the sorption–desorption CO_2_ isotherms are provided.

The sorption isotherm shows the sorbed volume of CO_2_ (V_STP_) reduced to standard temperature and pressure (STP) [34] vs. the relative pressure p/p_0_, where p is the equilibrium pressure and p_0_ is the saturated vapor pressure of the adsorbate. The value of CO_2_ uptake is 0.54 mmol/g, according to the isotherm data (at 1 bar). In Figure 9b, the corresponding DR plot [log V vs. log^2^(p_0_/p)] is shown. According to the DR method, the SV value is 0.104 cm^3^/g and the adsorption energy value is 18.7 kJ/mol. As found using the NL-DFT method, the SV value is 0.046 cm^3^/g and the SSA value reaches 123 m^2^/g for micropores. The difference in SV values for the DR and NL-DFT methods is expected and related to the different calculation approaches and micropore ranges for these methods. The pore size distributions based on the NL-DFT method for SSA (dV/dD vs. pore size (D), Figure 9c) and SV (dV/dD vs. pore size (D), Figure 9d) reveal the microporosity of the sample. A distinct peak at ~0.60 nm, as well as two lower peaks at 0.7–0.9 nm, are observed.

**Figure 10:**
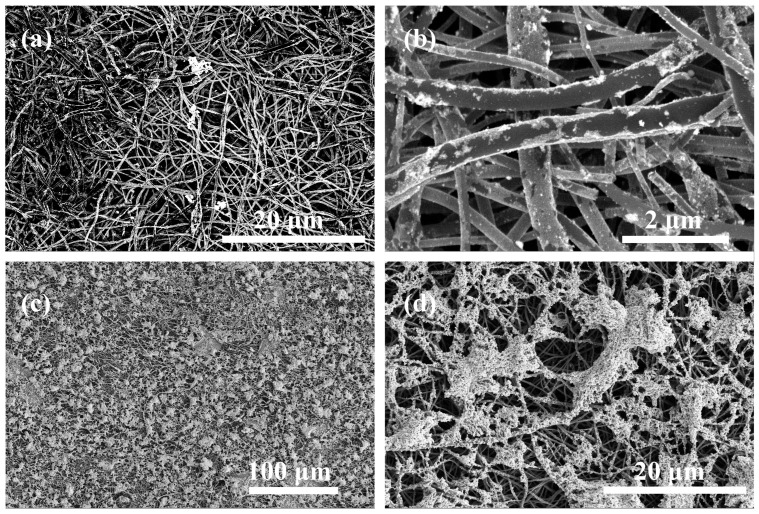
SEM images of (**a**,**b**) Pt/CNF and (**c**,**d**) PBI-4MeO-6F/Pt/CNF.

Scanning electron microscopy (SEM) was applied for the non-modified Pt/CNF anode (Figure 10a,b) and for the anode treated with 0.1 wt.% of PA-4MeO-6F in hexafluoroisopropanol with the following thermal cyclization at 300 °C (Figure 10c,d).

**Figure 11:**
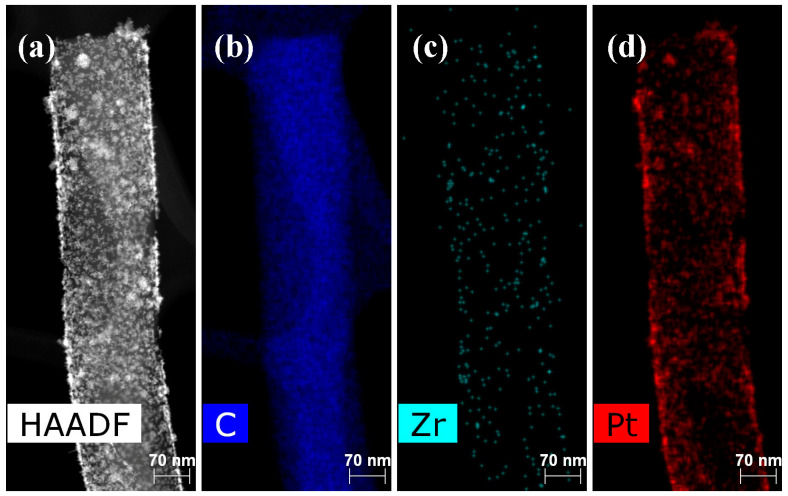
(**a**) HAADF STEM of Pt/CNF and the corresponding elemental distribution maps for (**b**) C, (**c**) Zr, and (**d**) Pt.

**Figure 12:**
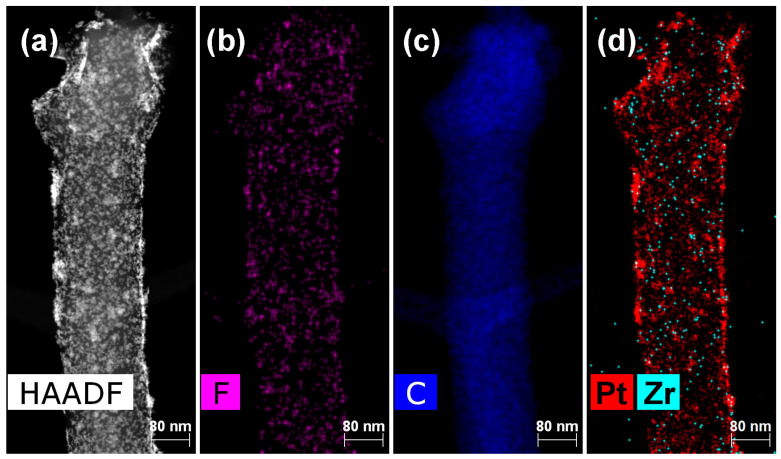
(**a**) HAADF STEM of PBI-4MeO-6F/Pt/CNF and the corresponding elemental distribution maps for (**b**) F, (**c**) C, and (**d**) Pt and Zr.

The HAADF STEM images and the corresponding elemental distribution maps for the non-modified Pt/CNF anode and for the anode treated with 0.1 wt.% of PA-4MeO-6F in hexafluoroisopropanol following thermal cyclization at 300 °C, i.e., the PBI-4MeO-6F/Pt/CNF anode, are shown in Figure 11 and Figure 12.

The Pt/CNF sample is characterized by a uniform distribution of Pt and Zr elements. In an earlier study [24], we have shown that, after the two steps of heat treatment, oxidation in air at 250 °C for 2 h and pyrolysis under vacuum at 1000 °C for 2 h, Zr appeared in the electrode in the form of ZrO_x_ distributed homogeneously throughout the nanofibers, as was confirmed using XPS and electron microscopy. Due to interaction with phosphoric acid, the formation of acid sites (zirconium hydrogen phosphate) is supposed to lead to an improvement in proton transport.

A layer of the PBI-4MeO-6F porous polymer is present on the surface of the PBI-4MeO-6F/Pt/CNF sample. Its presence is confirmed by the corresponding elemental distribution map, which shows a uniform distribution of F.

**Figure 13:**
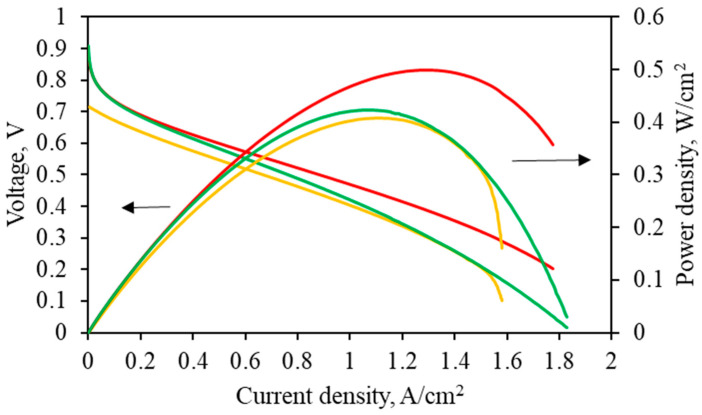
Polarization and power density curves for HT-PEMFC MEA at 180 °C with different anodes: Pt/CNF (yellow); PBI-O-PhT-P/Pt/CNF (green); and PBI-4MeO-6F/Pt/CNF (red).

The method of cyclic voltammetry (CV) was applied to the anode materials before and after polymer deposition (Figure S6). It appears that the value of the electrochemically active specific surface area (ECSA) of platinum decreases slightly for the modified sample but is still similar to the unmodified one. Recently [35,36], we have reported the HT-PEMFC MEA operation with polyheteroarylene-based CNF anodes. The most recent electrode materials and challenges related to HT-PEMFC were recently reviewed [37,38]. Therefore, here, we tried to apply the obtained anode materials in HT-PEMFC MEA. It was found that the MEA performance for the modified anodes (PBI-4MeO-6F/Pt/CNF) is higher than for the Pt/CNF anode, and even higher compared with the anode obtained previously [25], modified by PBI-O-PhT-P, i.e., the PBI-O-PhT-P/Pt/CNF anode (Figure 13).

As seen from Figure 13, for HT-PEMFC MEA with the PBI-4MeO-6F/Pt/CNF anode, the power density maximum reaches 500 mW/cm^2^ at 1.3 A/cm^2^ at 180 °C.

## 3. Materials and Methods

### 3.1. Materials

All chemicals, 1,5-dichloro-2,4-dinitrobenzene, 3,5-dimethoxyaniline, 4,4′-(hexafluoroisopropylidene)bis(benzoic acid chloride), *N*,*N*-dimethylacetamide, *N*-methylpyrrolidone (NMP), hydrazine hydrate, Pd/C (10%), phosphoric acid (85%), PAN, zirconium (IV) chloride were obtained from Acros Organics (Thermo Fisher Scientific, Waltham, MA, USA) and used as received without additional purification.

### 3.2. Synthesis

#### 3.2.1. Synthesis of *N*^1^,*N*^5^-bis(3,5-dimethoxyphenyl)-4,6-dinitro-1,3-benzenediamine

1,5-dichloro-2,4-dinitrobenzene (4.74 g, 0.02 mol) was added in small portions to a solution of 3,5-dimethoxyaniline (6.60 g, 0.043 mol) in DMA (15 mL) and triethylamine (6.5 mL) at 60 °C. The mixture was stirred for 8 h and then precipitated with ethanol (100 mL) and filtered to obtain 8.35 g (89%) of the product. M.p. 180–182 °C. Elemental analysis, %: calc. C 56.17; H 4.71; N 11.91. C_22_H_22_N_4_O_8_; M 470.44 g/mol. Found: C 56.47; H 4.81; N 11.84.

#### 3.2.2. Synthesis of *N*^1^,*N*^5^-bis(3,5-dimethoxyphenyl)-1,2,4,5-benzenetetramine

*N*^1^,*N*^5^-bis(3,5-methoxyphenyl)-4,6-dinitro-1,3-benzenediamine (7.6 g, 16.2 mmol) was mixed with ethanol (100 mL) and hydrogenated in 400 mL autoclave with 10% Pd/C catalyst (0.6 g) for 8 h at a pressure of 80 bar at 80–90 °C. The obtained solution was passed through silica gel and concentrated to 100 mL of total volume using a rotary evaporator then cooled until the precipitation of the product. The product was filtered and dried to obtain 5.42 g (81.5%). M.p. 166–168 °C. Elemental analysis: calc. C 64.38; H 6.38; N 13.65. C_22_H_26_N_4_O_4_. M 410.47 g/mol. Found C 64.49; H 6.46; N 13.56. ^1^H NMR (300 MHz, DMSO-d_6_**)** δ 6.84(s, 2H), 6.56(s, 1H), 6.16(s, 1H), 5.75(s, 4H), 5.74(s, 2H), 4.46(s, 4H), 3.61(s, 12H) (Figure S1).

#### 3.2.3. Synthesis of Polyamide PA-4MeO-6F and PBI-4MeO-6F

4,4′-(Hexafluoroisopropylidene)bis(benzoic acid chloride) (0.8585 g, 0.002 mol) was gradually added to a solution of *N*^1^,*N*^5^-bis(3,5-dimethoxyphenyl)-1,2,4,5-benzenetetramine (0.8210 g, 0.002 mol) in 3.5 mL of DMAc (or NMP) at room temperature (or −10 °C for 1 h). The mixture was then stirred under argon flow for 24 h. The PA-4MeO-6F films were prepared by casting 10 wt.% polymer reaction solutions onto a glass substrate. The films were dried at 60 °C overnight and then peeled off via immersion in water. A part of the solution was precipitated in methanol, filtered, and dried to obtain samples for further analysis. Intrinsic viscosity [η] 0.19–0.69 dL/g (25 °C, NMP). GPLC (NMP): M_n_ 31–38 kg/mol; M_w_ 50–78 kg mol^−1^ (PDI of 2.05–2.38). ^1^H and ^19^F NMR, (300 MHz, DMSO-*d*_6_) δ 9.85 (s, 1H), 8.11 (d, 2H), 8.09 (d, 2H), 7.97 (d, 2H), 7.94 (d, 2H), 7.89 (s, 2H), 7.57 (s, 2H), 7.28 (s, 1H), 6.14 (s, 4H), 6.00 (s, 2H), 3.70 (s, 12H) and −62.87 (s, 6F) (Figure S2).

Thermal heterocyclizaton of PA-4MeO-6F film was conducted at 300 °C under vacuum for 1 h with the formation of PBI-4MeO-6F.

### 3.3. Physical and Physico-Chemical Methods

The molecular weights of samples were measured using gel permeation chromatography (GPC) and a Knauer Smartline system (Knauer, Berlin, Germany) with a refractometric detector and Phenomenex Phenogel (5 µm) column. 0.03 M LiCl solution in NMP was used as an eluent with a flow rate of 1.0 mL/min at 25 °C. Calibration was performed using polystyrene standards.

Thermogravimetric analysis (TGA) and differential thermal analysis (DTA) measurements were performed on a Derivatograph-C (MOM Szerviz, Budapest, Hungary) at a heating rate of 10 K/min in air. The weight of the samples was ~12 mg.

The mechanical properties of the polymers for the polymer films were obtained using a 2166 R-5 tensile strength setup (Tochpribor, Ivanovo, Russia) in the tension mode at room temperature and ambient pressure with a crosshead speed of 10^−4^ m/s.

The CV measurements were performed at room temperature in a three-electrode cell with separated compartments to obtain the ECSA of platinum. Platinum wire and Ag/AgCl-saturated KCl (0.2 V vs. SHE) were applied as counter and reference electrodes, respectively. A polished graphite disk 1.6 cm^2^ in diameter in a PTFE holder was applied as a working electrode. Inks of electrocatalyst were prepared by ultrasonically dispersing 2–3 mg of an electrocatalyst in 0.4 mL of aqueous solution containing 0.01 mL of 5 wt.% Nafion solution and 0.2 mL of isopropanol. An electrocatalyst layer coating the surface of the disk electrode by repeatedly placing aliquots of the electrocatalyst ink with intermediate drying (60 °C). The ink (100 µL) was dispersed onto the disk electrode surface, fifty cycles of voltammetry at 50 mV/s were conducted, and the last cycle was examined. Platinum ECSA was evaluated through the integration of the hydrogen adsorption/desorption areas of the CV assuming 0.21 mC/cm^2^_Pt_.

Nuclear magnetic resonance (NMR) spectra of the studied compounds were recorded on a Bruker Avance 300 spectrometer (Bruker, Billerica, MA, USA).

Fourier-transform infrared spectroscopy (FTIR) of individual compounds in KBr pellets and polymer films was performed in absorbance mode using an InfraRed Bruker Tensor 37 FTIR spectrometer (Bruker, Billerica, MA, USA) using a spectral range of 5000–500 cm^−1^.

The CO_2_ sorption–desorption isotherms were obtained in a range of 10^−3^–1 bar at 273.15 K on a 3P Micro 200 Surface Area and Pore Size Analyzer (3P Instruments, Odelzhausen, Germany). The specific volume (SV) and specific surface area (SSA) values were found using the non-local density functional theory (NLDFT) method using NovaWin, version 11.04, Quantachrome Instruments (Boynton Beach, FL, USA). The Dubinin–Radushkevich method was applied to find SV and adsorption energy. The value of the CO_2_ uptake was determined at standard temperature and pressure (STP, according to IUPAC) of 273.15 K and 100 kPa [34]. The CO_2_ cross-sectional area was considered to be 0.210 nm^2^; the affinity coefficient β was taken as 0.35 [39]; saturated vapor pressure of the adsorbate (p_0_) and adsorbed CO_2_ density were taken as 3.485 MPa and 1.044 g/cm^3^, respectively.

The structure of the composite electrospun Pt/CNF and PBI-4MeO-6F/Pt/CNF were investigated with scanning electron microscopy (SEM) using a FEI Scios microscope (Hillsboro, OR, USA) and scanning transmission electron microscopy with a high-angle annular dark-field detector (HAADF STEM) and energy-dispersive X-ray spectroscopy (EDX) elemental mapping using a Thermo Fisher Scientific Osiris (Waltham, MA, USA) equipped with a high-angle annular dark field (HAADF) detector and Super-X EDX detection system based on Silicon Drift Detector (SDD) technology. For electron microscopy studies, the samples of CNF were dispersed for ~30 min in acetone to separate fibers using an ultrasonic bath. Electron microscope images were analyzed using Digital Micrograph Gatan GMS 3 (Pleasanton, CA, USA), Siemens AG TIA 16 (Munich, Germany), JEMS software (EMS Java version 2004, P. Stadelmann JEMS, EPFL, https://www.jems-swiss.ch/, accessed on 24 April 2024, Lausanne, Switzerland), and Bruker Esprit 2 (Billerica, MA, USA).

### 3.4. Electrode Preparation

#### 3.4.1. Electrospinning

The composite PAN-based nanofiber mats were obtained according to Nanospider^TM^ technology using the needle-free electrospinning method from a free surface. The process was performed using an NS Lab Nanospider^TM^ setup (Elmarco, Liberec, Czechia) at a relative humidity of 8% and voltage of 69 kV with a distance between electrodes of 190 mm. The electrospinning polymer solution contained 3.25 g of PAN (M_w_ 150 kDa), 0.1 g of Vulcan XC72 carbon black (~3 wt.% relative to PAN), and 0.03 g of zirconium (IV) chloride well dispersed in 50 mL of DMF in an ultrasonic bath for 3 h. As a result, PAN/Vulc/Zr composite nanofibers were obtained in the form of a self-supporting mat.

#### 3.4.2. Stabilization, Zinc Deposition and Pyrolysis

PAN/Vulc/Zr nanofiber mat was stabilized (oxidized) at 250 °C in the air for 2 h in a Binder MDL 115 heating chamber (Tuttlingen, Germany). The obtained material (PAN/Vulc/Zr/Ni-250) was immersed in 0.5 wt.% of Zn(NO_3_)_2_ solution in a water–isopropanol mixture (1:3 *v*/*v*) for ~24 h and dried for 2 h at 100 °C. Then, the sample was pyrolyzed at 1000 °C for 2 h under vacuum at a heating rate of 3 °C min^−1^ in a Carbolite (CTF 12/80/700) vacuum oven (Hope Valley, UK). As a result, the self-supporting CNF mat (PAN/Vulc/Zr-250/Zn-1000) was obtained.

#### 3.4.3. Platinum Deposition

Platinum deposition on the CNF (PAN/Vulc/Zr-250/Zn-1000) mats with an area of 6.76 cm^2^ was carried out separately for each mat in 10 mL of water containing the calculated amount of H_2_[PtCl_6_]·6H_2_O as a source of platinum to obtain anode electrocatalysts with a Pt load of 1.2 mg_Pt_/cm^2^. Formic acid (0.5 g) was used as a reducing agent, and the mixture was stored for 3 days at room temperature. The resulting Pt/CNF was thoroughly washed with distilled water and dried at 100 °C for 2 h under vacuum.

#### 3.4.4. Polymer Deposition

The self-phosphorylated proton-conducting polymer PBI-4MeO-6F was deposited onto Pt/CNF mats in two steps. First, the immersing of Pt/CNF into a 0.1 wt.% solution of PA-4MeO-6F in hexafluoroisopropanol for 15 min. Second, air drying the PA-4MeO-6F-coated mats when converted into PBI-4MeO-6F anodes using heat treatment and under vacuum for 1 h at 300 °C. As a result, an electrode, PBI-4MeO-6F/Pt/CNF, was prepared.

### 3.5. Gas Permeability

The permeability values for H_2_, N_2_, and O_2_ gases for the PBI-4MeO-6F and PBI-O-PhT-P film samples were obtained using the integral barometric method at 35 °C on a MKS Baratron setup (MKS Instruments, Andover, MA, USA). The pressure above the membrane was maintained at 1–5 bar, while the pressure below the membrane was ~0.16 mbar. The permeability coefficients were determined from a steady-state gas permeation experiment [40,41]. The measurement error for the gas permeability is 5%.

### 3.6. HT-PEM Fuel Cell Operation

The performance of the membrane–electrode assembly with the PBI-O-PhT membrane (a membrane, previously developed in our group [18,42,43]) was studied using a standard test fuel cell with two graphite flow field plates (Arbin Instruments, College Station, TX, USA) at 180 °C. Standard gas diffusion cathodes Celtec^®^-P Series 1000 MEA [44] were used to build the MEA. The MEA was completed by placing the PBI-O-PhT membrane between the Celtec^®^ gas diffusion cathode and the CNF-based anode, developed in the current study. Up to ~25% contraction of the gas diffusion electrodes was achieved using the polytetrafluoroethylene gaskets of the required thickness.

The working area of the MEA was 5 cm^2^. MEA polarization curves were recorded at 180 °C and ambient pressure using an Elins P-200X Potentiostat (Electrochemical Instruments, Chernogolovka, Russia). The anode was supplied with H_2_ at a rate of 100 mL/min. The cathode was supplied with atmospheric air at a rate of 800 mL/min. In voltammetry measurements, FC voltage was scanned at a rate of 5 mV/s in the range of 0.95–0.1 V. Polarization curves were stabilized after 2–3 h of the cell voltage cycling.

## 4. Conclusions

A novel self-phosphorylating polybenzimidazole of the 6F family (PBI-4MeO-6F) has been used as a surface modifier for the carbon nanofiber-based anode to improve the proton conductivity of the Pt/CNF anode, as well as triple-phase boundary and gas transport. The polymer showed excellent gas permeability values and is promising for application as an electrode coating. The anodes were tested in MEA for hydrogen/air HT-PEM fuel cells. The obtained coating ensures a continuous interface between the anode and proton-conducting membrane and leads to improved performance of the HT-PEM fuel cell MEA. It has been shown that the application of the unique self-phosphorylating PBI of the 6F family to coat the CNF anodes results in an enhancement of the HT-PEM fuel cell performance compared with the previously studied anodes when the PBI-O-PhT-P polymer was used as a surface modifier. At 180 °C, the resulting power density maximum for the HT-PEMFC MEA with the PBI-4MeO-6F/Pt/CNF anode reaches 500 mW/cm^2^ (at 1.3 A/cm^2^).

## Figures and Tables

**Table 1:** Additional data on the PA-4MeO-6F synthesis.

Solvent	C, mol/L	T, °C	Yeild, %	[η], dL/g *	M_w_, kg/mol	M_n_, kg/mol	M_w_/M_n_
DMA	1.3	25	97	0.21	68	31	2.19
DMA/Et_3_N	1.3	25	96	0.19	50	21	2.38
NMP	1.0	−10	99	0.69	78	38	2.05

* in NMP at 25 °C.

**Table 2:** Gas permeability of the PBI-4MeO-6F and PBI-O-PhT-P polymer films for hydrogen, oxygen, and nitrogen.

Polymer	H_2_, Barrer	O_2_, Barrer	N_2_, Barrer
PBI-4MeO-6F	41.1	7.15	1.97
PBI-O-PhT-P	9.66	0.88	0.22

In SI, 1 Barrer = 3.35 × 10^−16^ (mol·m)/(m^2^·s·Pa) [32].

## Data Availability

The authors confirm that the data supporting the findings of this study are available within the article and its Supplementary Materials.

## Supplementary Materials

The following supporting information can be downloaded at: https://www.mdpi.com/article/10.3390/ijms25116001/s1.

## References

[1] Peinemann K.-V., Nunes S.P. (2008). Membranes for Energy Conversion.

[2] Zhang J. (2008). PEM Fuel Cell Electrocatalysts and Catalyst Layers, Fundamentals and Applications.

[3] Aili D., Henkensmeier D., Martin S., Singh B., Hu Y., Jensen J.O., Cleemann L.N., Li Q. (2020). Polybenzimidazole-Based High-Temperature Polymer Electrolyte Membrane Fuel Cells: New Insights and Recent Progress. Electrochem. Energy Rev..

[4] Li Q., Aili D., Hjuler H.A., Jensen J.O. (2016). High Temperature Polymer Electrolyte Membrane Fuel Cells, Approaches, Status and Perspectives.

[5] Moorthy S., Sivasubramanian G., Kannaiyan D., Deivanayagam P. (2023). Neoteric advancements in polybenzimidazole based polymer electrolytes for high-temperature proton exchange membrane fuel cells—A versatile review. Int. J. Hydrogen Energy.

[6] Li Q., He R., Jensen J.O., Bjerrum N.J. (2004). PBI-Based Polymer Membranes for High Temperature Fuel Cells–Preparation, Characterization and Fuel Cell Demonstration. Fuel Cells.

[7] Escorihuela J., Olvera-Mancilla J., Alexandrova L., del Castillo L.F., Compañ V. (2020). Recent Progress in the Development of Composite Membranes Based on Polybenzimidazole for High Temperature Proton Exchange Membrane (PEM) Fuel Cell Applications. Polymers.

[8] Kalathil A., Raghavan A., Kandasubramanian B. (2019). Polymer Fuel Cell Based on Polybenzimidazole Membrane: A Review. Polym.-Plast. Technol. Mater..

[9] Pingitore A.T., Molleo M., Schmidt T.J., Benicewicz B.C., Lipman T., Weber A. (2019). Polybenzimidazole Fuel Cell Technology: Theory, Performance, and Applications. Fuel Cells and Hydrogen Production.

[10] Haque M.A., Sulong A.B., Loh K.S., Majlan E.H., Husaini T., Rosli R.E. (2017). Acid doped polybenzimidazoles based membrane electrode assembly for high temperature proton exchange membrane fuel cell: A review. Int. J. Hydrogen Energy.

[11] Quartarone E., Angioni S., Mustarelli P. (2017). Polymer and Composite Membranes for Proton-Conducting, High-Temperature Fuel Cells: A Critical Review. Materials.

[12] Myles T., Bonville L., Maric R. (2017). Catalyst, Membrane, Free Electrolyte Challenges, and Pathways to Resolutions in High Temperature Polymer Electrolyte Membrane Fuel Cells. Catalysts.

[13] Zeis R. (2015). Materials and characterization techniques for high-temperature polymer electrolyte membrane fuel cells. Beilstein J. Nanotechnol..

[14] Araya S.S., Zhou F., Liso V., Sahlin S.L., Vang J.R., Thomas S., Gao X., Jeppesen C., Kaer S.K. (2016). A comprehensive review of PBI-based high temperature PEM fuel cells. Int. J. Hydrogen Energy.

[15] Lim K.H., Lee A.S., Atanasov V., Jochen K., Park E.J., Adhikari S., Maurya S., Manriquez L.D., Jung J., Fujimoto C. (2022). Protonated phosphonic acid electrodes for high power heavy-duty vehicle fuel cells. Nat. Energy.

[16] Vogel H., Marvel C.S. (1961). Polybenzimidazoles, new thermally stable polymers. J. Polym. Sci..

[17] Ponomarev I.I., Razorenov D.Y., Ponomarev I.I., Volkova Y.A., Skupov K.M. (2014). Synthesis and studies of polybenzimidazoles for high temperature fuel cells. Russ. J. Electrochem..

[18] Kondratenko M.S., Ponomarev I.I., Gallyamov M.O., Razorenov D.Y., Volkova Y.A., Kharitonova E.P., Khokhlov A.R. (2013). Novel composite Zr/PBI-O-PhT membranes for HT-PEFC applications. Beilstein J. Nanotechnol..

[19] Ponomarev I.I., Skupov K.M., Modestov A.D., Lysova A.A., Ponomarev I.I., Vtyurina E.S. (2022). Cardo Polybenzimidazole (PBI-O-PhT) Based Membrane Reinforced with m-polybenzimidazole Electrospun Nanofiber Mat for HT-PEM Fuel Cell Applications. Membranes.

[20] Ponomarev I.I., Goryunov E.I., Petrovskii P.V., Ponomarev I.I., Volkova Y.A., Razorenov D.Y., Khokhlov A.R. (2009). Synthesis of new monomer 3,3′-diamino-4,4′-bis{p-[(diethoxyphosphoryl)methyl]phenylamino}diphenyl sulfone and polybenzimidazoles on its basis. Dokl. Chem..

[21] Ponomarev I.I., Ponomarev I.I., Goryunov E.I., Volkova Y.A., Razorenov D.Y., Starikova Z.A., Blagodatskikh I.V., Buzin M.I., Khokhlov A.R. (2012). Chemical modification of cardo poly(benzimidazole) using “click” reaction for membranes of high-temperature hydrogen fuel cells. Dokl. Chem..

[22] Ponomarev I.I., Razorenov D.Y., Ponomarev I.I., Volkova Y.A., Skupov K.M., Lysova A.A., Yaroslavtsev A.B., Modestov A.D., Buzin M.I., Klemenkova Z.S. (2021). Polybenzimidazoles via polyamidation: A more environmentally safe process to proton conducting membrane for hydrogen HT-PEM fuel cell. Eur. Polym. J..

[23] Ponomarev I.I., Razorenov D.Y., Skupov K.M., Ponomarev I.I., Volkova Y.A., Lyssenko K.A., Lysova A.A., Vtyurina E.S., Buzin M.I., Klemenkova Z.S. (2023). Self-Phosphorylated Polybenzimidazole: An Environmentally Friendly and Economical Approach for Hydrogen/Air High-Temperature Polymer-Electrolyte Membrane Fuel Cells. Membranes.

[24] Ponomarev I.I., Skupov K.M., Naumkin A.V., Basu V.G., Zhigalina O.M., Razorenov D.Y., Ponomarev I.I., Volkova Y.A. (2019). Probing of complex carbon nanofiber paper as gas-diffusion electrode for high temperature polymer electrolyte membrane fuel cell. RSC Adv..

[25] Skupov K.M., Ponomarev I.I., Vtyurina E.S., Volkova Y.A., Ponomarev I.I., Zhigalina O.M., Khmelenin D.N., Cherkovskiy E.N., Modestov A.D. (2023). Proton-Conducting Polymer-Coated Carbon Nanofiber Mats for Pt-Anodes of High-Temperature Polymer-Electrolyte Membrane Fuel Cell. Membranes.

[26] Cetina-Mancilla E., González-Díaz M.O., Sulub-Sulub R., Zolotukhin M.G., González-Díaz A., Herrera-Kao W., Ruiz-Trevino F.A., Aguilar-Vega M. (2022). Aging resistant, fluorinated aromatic polymers with ladderized, rigid kink-structured backbones for gas separations. J. Membr. Sci..

[27] Ghanem B.S., McKeown N.B., Budd P.M., Al-Harbi N.M., Fritsch D., Heinrich K., Starannikova L., Tokarev A., Yampolskii Y. (2009). Synthesis, characterization, and gas permeation properties of a novel group of polymers with intrinsic microporosity: PIM-polyimides. Macromolecules.

[28] Han S.H., Lee J.E., Lee K.-J., Park H.B., Lee Y.M. (2010). Highly gas permeable and microporous polybenzimidazole membrane by thermal rearrangement. J. Membr. Sci..

[29] Kumbharkar S.C., Karadkar P.B., Kharul U.K. (2006). Enhancement of gas permeation properties of polybenzimidazoles by systematic structure architecture. J. Membr. Sci..

[30] Socrates G. (2004). Infrared and Raman Characteristic Group Frequencies: Tables and Charts.

[31] Lysova A.A., Ponomarev I.I., Skupov K.M., Vtyurina E.S., Lysov K.A., Yaroslavtsev A.B. (2022). Effect of Organo-Silanes Structure on the Properties of Silane-Crosslinked Membranes Based on Cardo Polybenzimidazole PBI-O-PhT. Membranes.

[32] Stern S.A. (1968). The “barrer” permeability unit. J. Polym. Sci. A-2 Polym. Phys..

[33] Lozano-Castello D., Cazorla-Amoros D., Linares-Solano A. (2004). Usefulness of CO_2_ adsorption at 273 K for the characterization of porous carbons. Carbon.

[34] Ewing M.B., Lilley T.H., Olofsson G.M., Ratzsch M.T., Somsen G. (1994). Standard quantities in chemical thermodynamics. Fugacities, activities and equilibrium constants for pure and mixed phases (IUPAC Recommendations 1994). Pure Appl. Chem..

[35] Skupov K.M., Vtyurina E.S., Ponomarev I.I., Ponomarev I.I., Aysin R.R. (2023). Prospective carbon nanofibers based on polymer of intrinsic microporosity (PIM-1): Pore structure regulation for higher carbon sequestration and renewable energy source applications. Polymer.

[36] Vtyurina E.S., Ponomarev I.I., Naumkin A.V., Bukalov S.S., Aysin R.R., Ponomarev I.I., Zhigalina O.M., Khmelenin D.N., Skupov K.M. (2024). Influence of the Polymer Precursor Structure on the Porosity of Carbon Nanofibers: Application as Electrode in High-Temperature Proton Exchange Membrane Fuel Cells. ACS Appl. Nano Mater..

[37] Seselj N., Alfaro S.M., Bompolaki E., Cleemann L.N., Torres T., Azizi K. (2023). Catalyst Development for High-Temperature Polymer Electrolyte Membrane Fuel Cell (HT-PEMFC) Applications. Adv. Mater..

[38] Zucconi A., Hack J., Stocker R., Suter T.A.M., Rettie A.J.E., Brett D.J.L. (2024). Challenges and opportunities for characterisation of high-temperature polymer electrolyte membrane fuel cells: A review. J. Mater. Chem. A.

[39] Linares-Solano A., Stoeckli F. (2005). Commentary on the paper “On the adsorption affinity coefficient of carbon dioxide in microporous carbons” by E.S. Bickford et al. (Carbon 2004; 42: 1867–71). Carbon.

[40] Mateucci S., Yampolskii Y., Freeman B.D., Pinnau I., Freeman B.D., Yampolskii Y., Pinnau I. (2006). Transport of Gases and Vapors in Glassy and Rubbery Polymers. Materials Science of Membranes for Gas and Vapor Separation.

[41] Koros W.J., Zimmerman C.M., Brady R.F.J. (2003). Transport and Barrier Properties. Comprehensive Desk Reference of Polymer Characterization and Analysis.

[42] Ponomarev I.I., Skupov K.M., Zhigalina O.M., Naumkin A.V., Modestov A.D., Basu V.G., Sufiyanova A.E., Razorenov D.Y., Ponomarev I.I. (2020). New Carbon Nanofiber Composite Materials Containing Lanthanides and Transition Metals Based on Electrospun Polyacrylonitrile for High Temperature Polymer Electrolyte Membrane Fuel Cell Cathodes. Polymers.

[43] Skupov K.M., Ponomarev I.I., Vol’fkovich Y.M., Modestov A.D., Ponomarev I.I., Volkova Y.A., Razorenov D.Y., Sosenkin V.E. (2020). The Effect of the Stabilization and Carbonization Temperatures on the Properties of Microporous Carbon Nanofiber Cathodes for Fuel Cells on Polybenzimidazole Membrane. Polym. Sci. Ser. C.

[44] Schmidt T.J., Baurmeister J. (2008). Properties of high-temperature PEFC Celtec^®^-P 1000 MEAs in start/stop operation mode. J. Power Sources.

