# Supplementary material for: Unique Self-Phosphorylating Polybenzimidazole of the 6F Family for HT-PEM Fuel Cell Application
Source: Int J Mol Sci. 2024 May 30;25(11):6001. doi: 10.3390/ijms25116001 (PMC11172766; doi:10.3390/ijms25116001)
Supplement: Supplementary file 1 [file ijms-25-06001-s001.zip › ijms-3006682-supplementary.pdf]

# Supplementary Materials

## Unique Self-Phosphorylating Polybenzimidazole of the 6F Family for HT-PEM Fuel Cell Application

Igor I. Ponomarev, Yulia A. Volkova, Kirill M. Skupov, Elizaveta S. Vtyurina, Ivan I. Ponomarev, Mikhail M. Ilyin, Roman Y. Nikiforov, Alexander Y. Alentiev, Olga M. Zhigalina, Dmitry N. Khmelenin, Tatyana V. Strelkova and Alexander D. Modestov

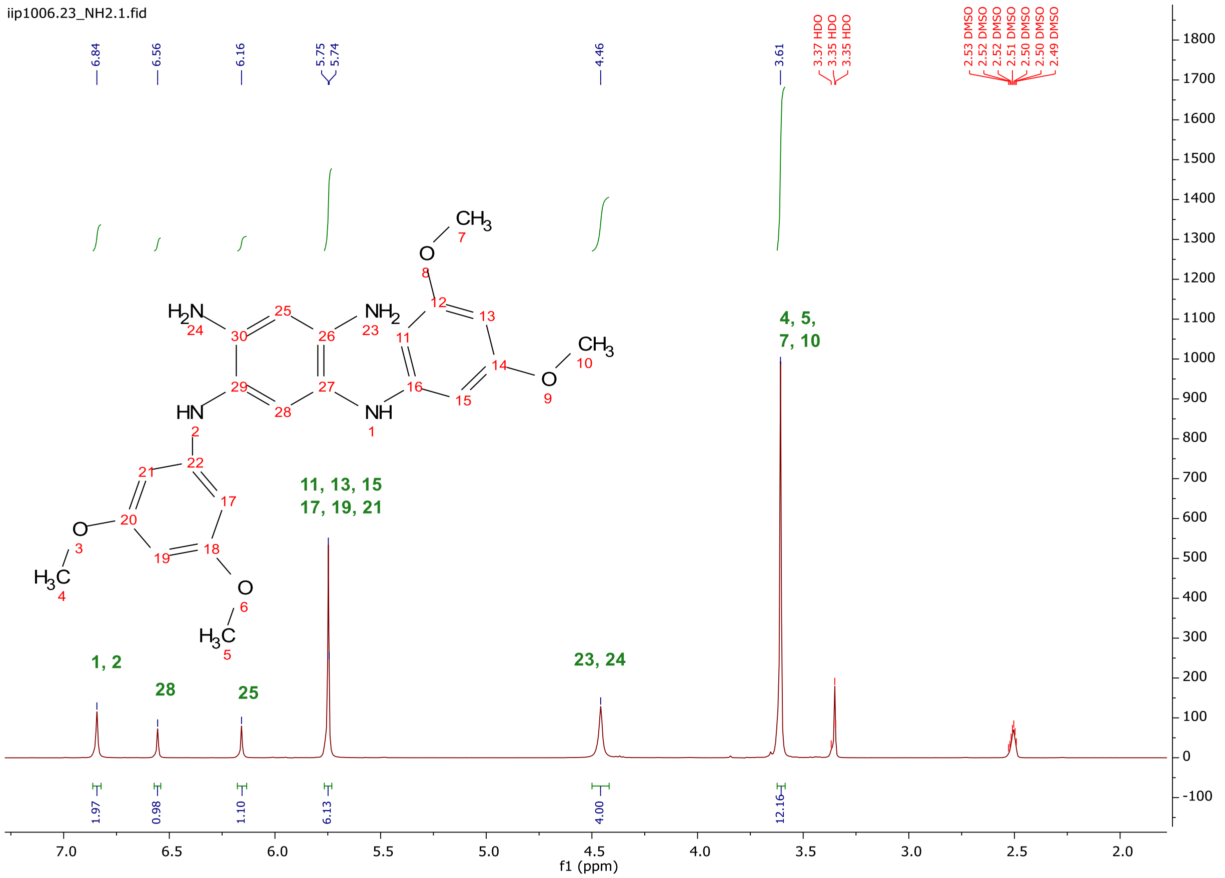


**Figure S1.** $^1$H NMR spectrum of N$^1$,N$^5$-bis(3,5-dimethoxyphenyl)-1,2,4,5-benzenetetramine (1)

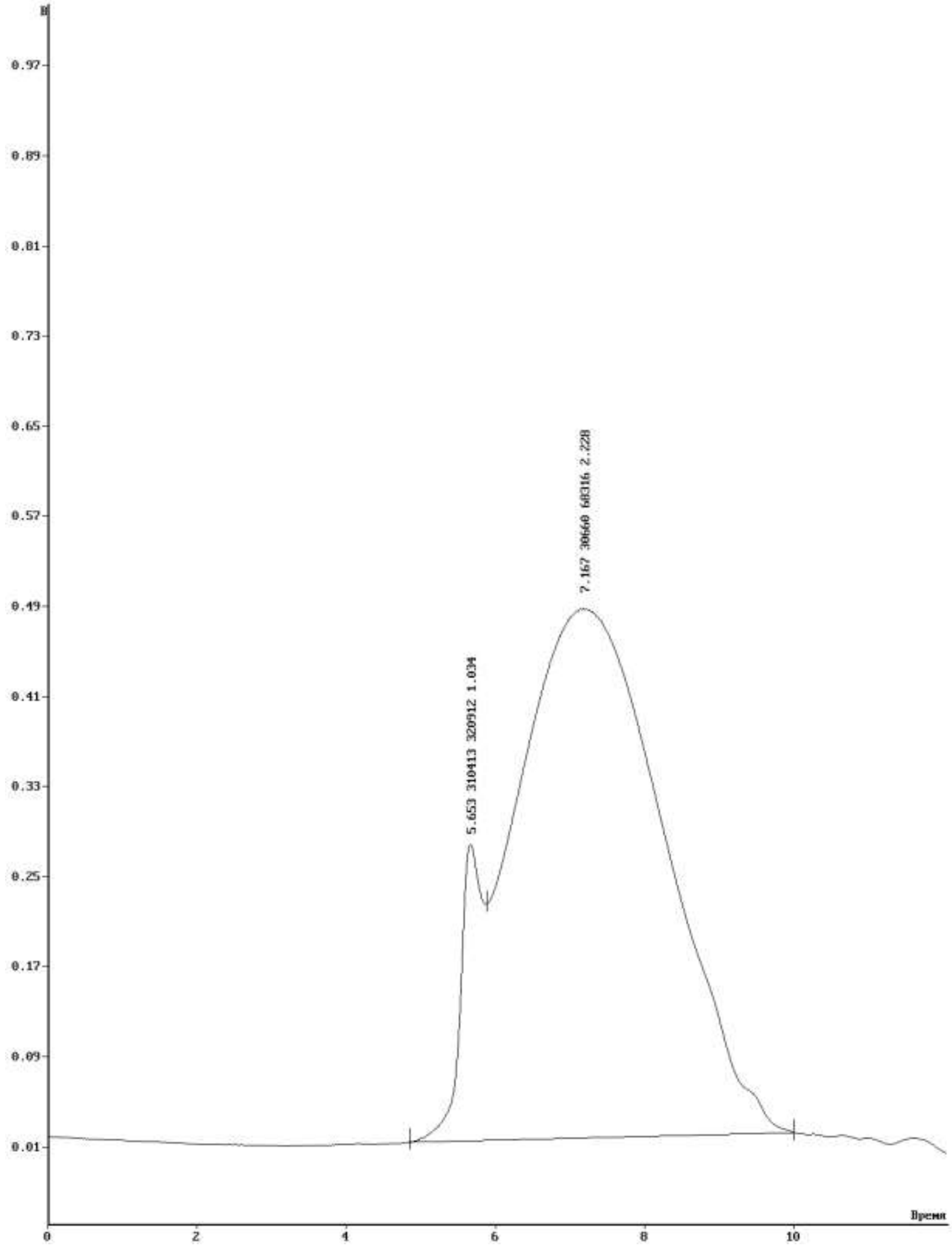


**Figure S2.** GPC curve for the PA-4MeO-6F polymer synthesized in DMA.

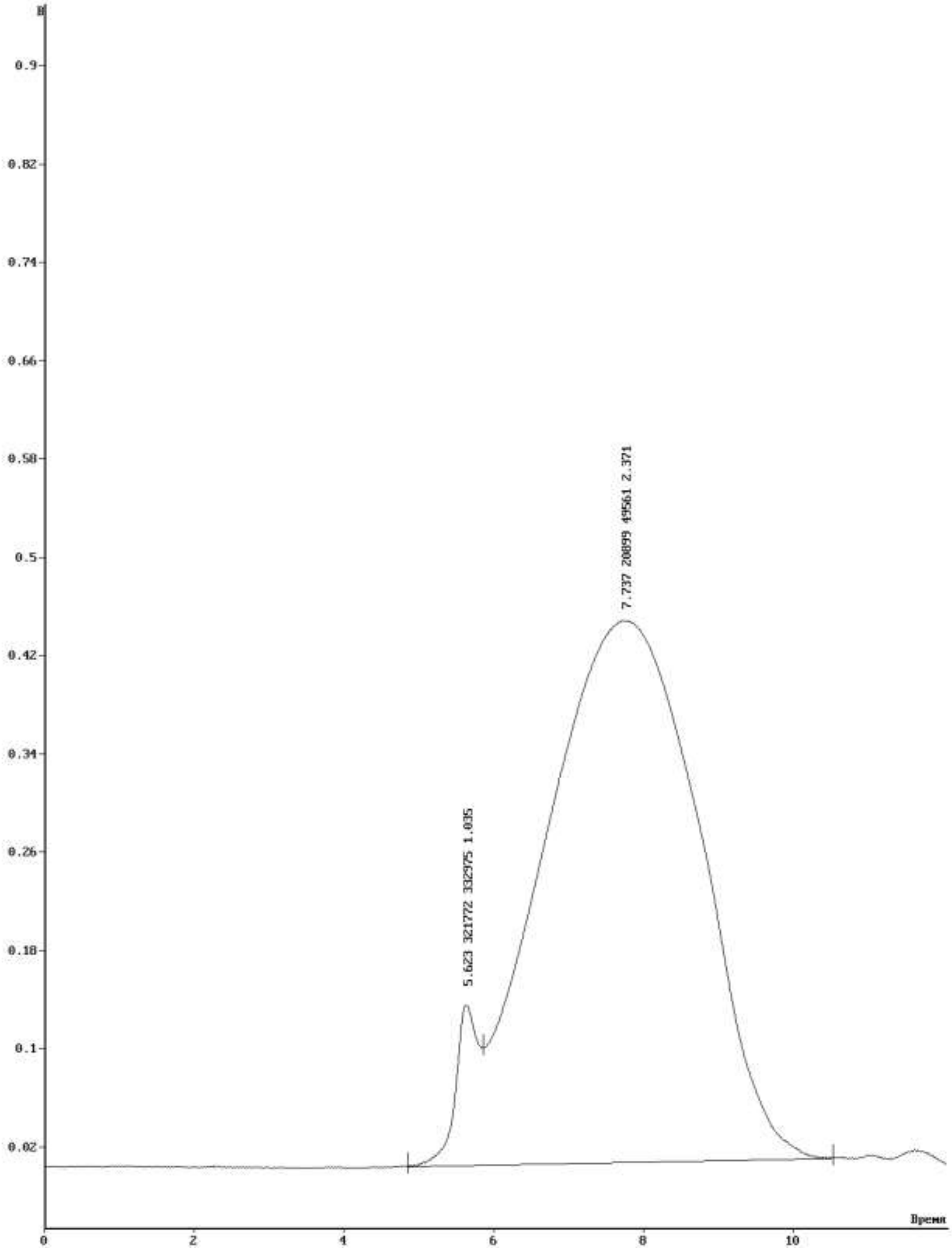


**Figure S3.** GPC curve for the PA-4MeO-6F polymer synthesized in DMA/$Et_3N$.

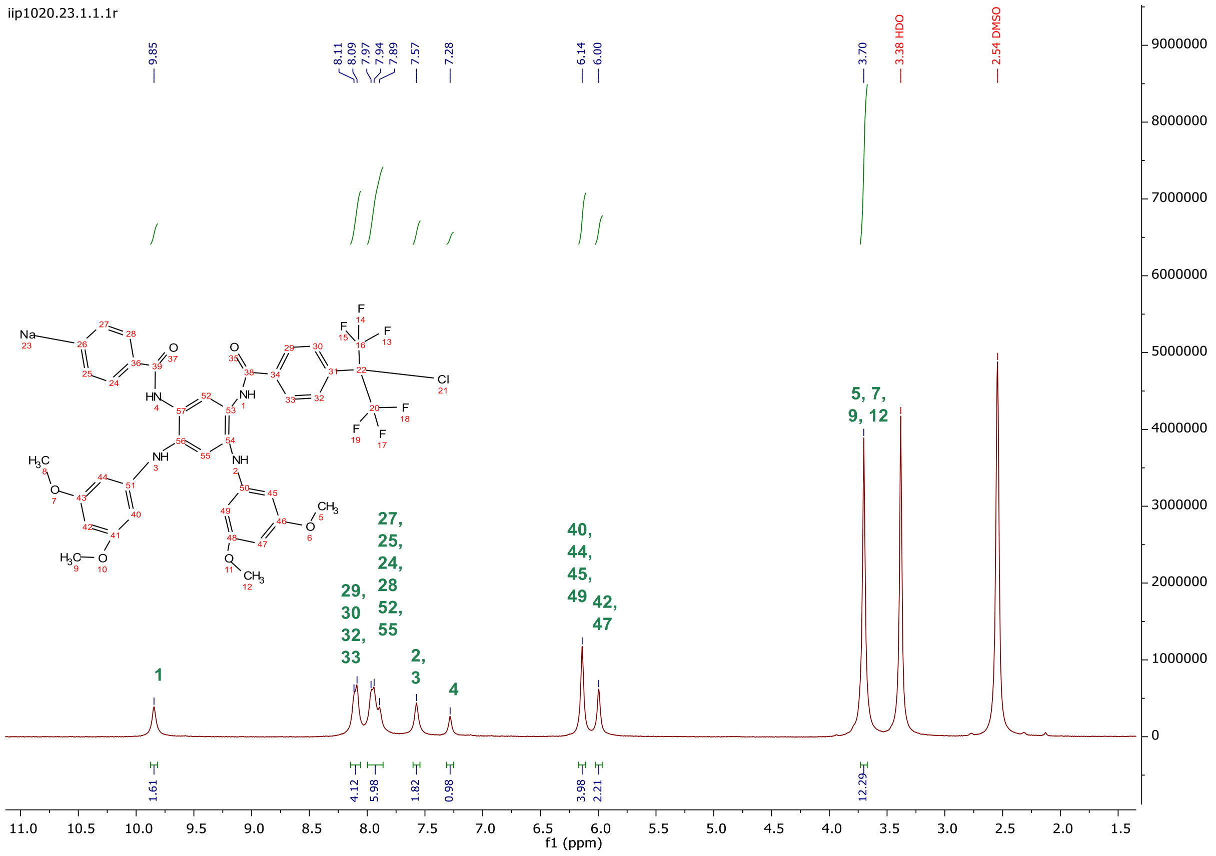


**Figure S4.** $^{1}H$ NMR spectrum of PA-4MeO-6F.

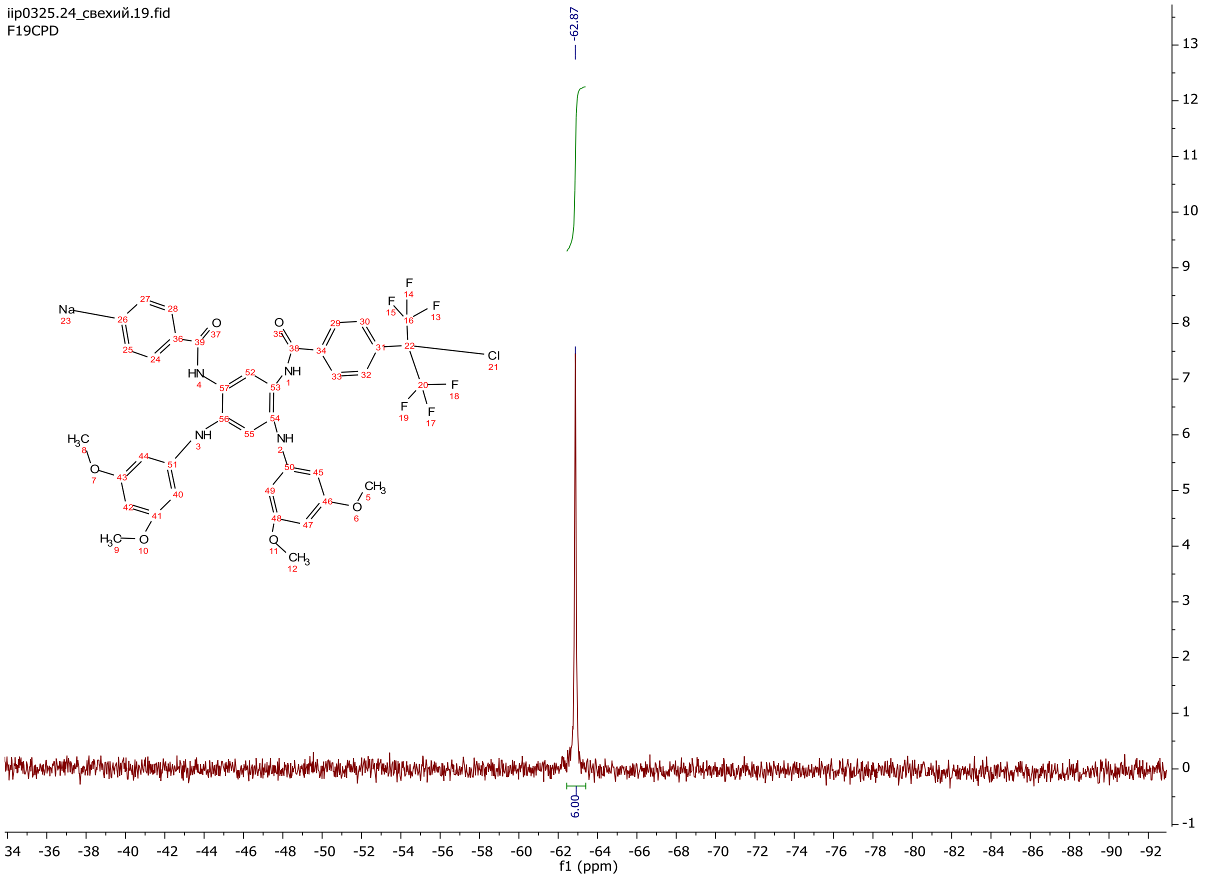


**Figure S5.** $^{19}F$ NMR spectrum of PA-4MeO-6F.

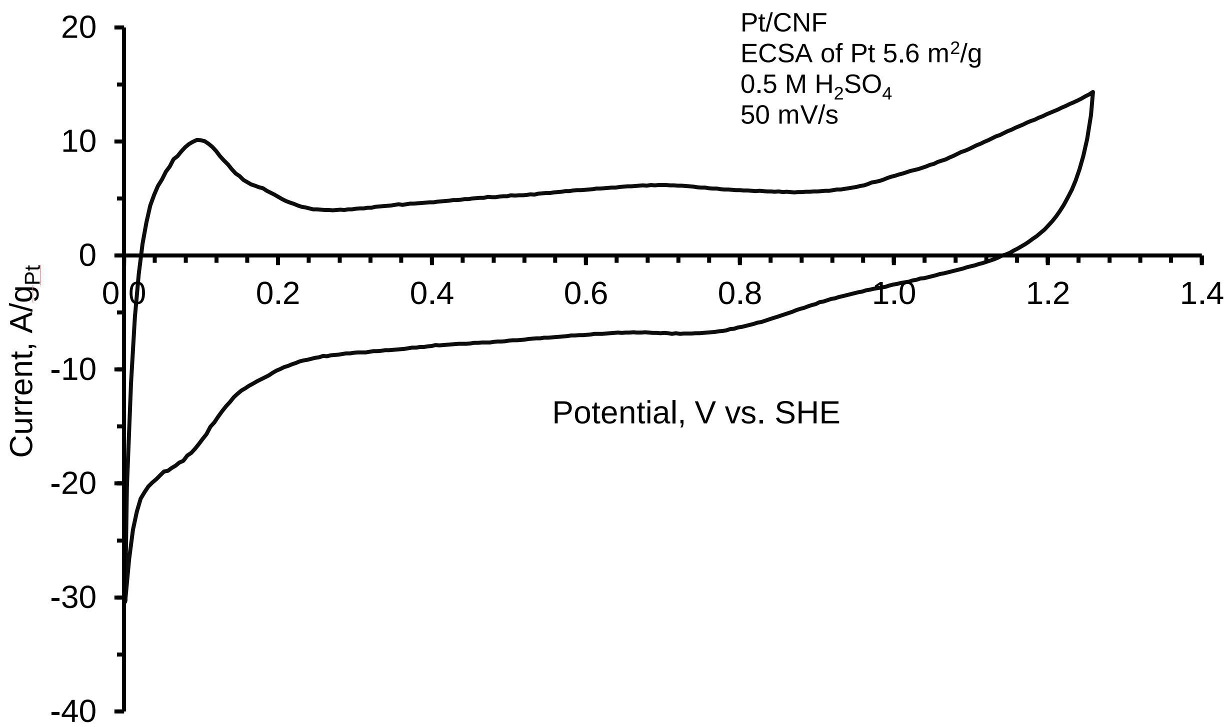


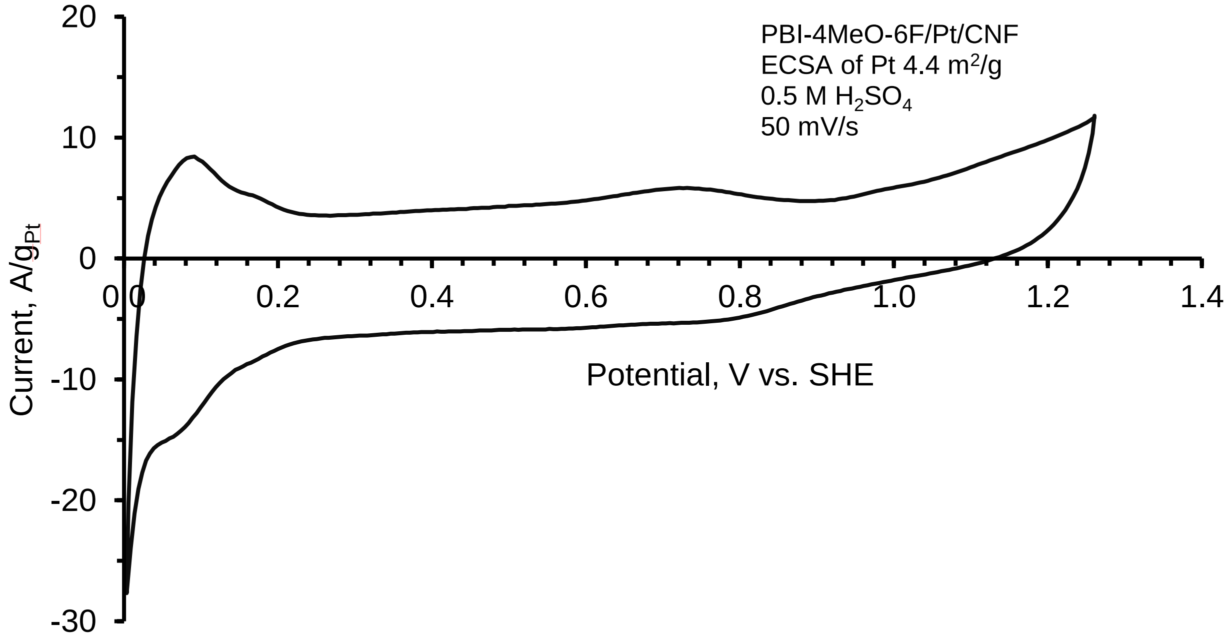


**Figure S6.** CV curves for Pt/CNF (top) and PBI-4MeO-6F/Pt/CNF (bottom).
